# A relational frame approach to perspective taking in persons with Borderline Personality Disorder

**DOI:** 10.1016/j.jcbs.2024.100777

**Published:** 2024-05-18

**Authors:** Carla J. Walton, Alison Rasmussen, Matthieu Villatte, Roger Vilardaga, Lauren Irwin, Rachel Rossiter

**Affiliations:** a Hunter New England Mental Health Service, Newcastle, NSW, 2300, Australia; b School of Medicine and Public Health, College of Health, Medicine and Wellbeing, University of Newcastle, Callaghan, NSW, 2308, Australia; c Hunter Medical Research Institute (HMRI), New Lambton, NSW, 2305, Australia; d School of Psychology, Faculty of Science, University of Newcastle, University Drive, Callaghan, NSW, 2308, Australia; e Private Practice, Seattle, USA; f Division of Public Health Sciences, Department of Implementation Science, Medical Center Boulevard \\ Winston-Salem, Wake Forest University Health Sciences, NC, 27157, USA; g School of Rural Medicine, Faculty of Science and Health, Charles Sturt University, Leeds Parade, Orange, NSW, 2800, Australia

**Keywords:** Perspective taking, Borderline Personality Disorder, Relational Frame Theory, Deictic frame

## Abstract

Perspective taking is important for effective interpersonal functioning. According to Relational Frame Theory (RFT), perspective taking is underpinned by deictic relational framing. It has been proposed that individuals with Borderline Personality Disorder (BPD) may have deficits in perspective taking. A mixed experimental design (*N* = 112) was used to assess whether individuals with a diagnosis of BPD displayed impaired perspective taking on a computerised RFT deictic relational task (DRT) and a self-report measure, compared to a control sample. There was no significant difference between groups on the computerised DRT. Within the clinical group, overall distress and relational distress were not found to be significantly associated with DRT performance or self-reported perspective taking. However, those with BPD self-reported significantly worse perspective taking ability compared to the control sample. This finding indicates a discrepancy between perceived perspective taking ability and direct perspective taking performance in persons with BPD.

Perspective taking is the ability to recognise and understand the thoughts, emotions, or behaviors of oneself and of others and is required for effective interpersonal functioning (Davis, 1994; Gerace et al., 2013; Healey & Grossman, 2018; Vilardaga, 2009a,b,c). It is also considered essential for human socialisation in terms of individuals being able to adjust their behavior as required (Kavanagh et al., 2020; Vilardaga & Hayes, 2012). Perspective taking research has primarily been conducted under the Theory of Mind (ToM) framework, where inferences are made about the intentions, emotions, and beliefs (i.e. mental states) of others to predict or interpret behavior (Barnes-Holmes et al., 2004; Baron-Cohen, 1997; Hendriks et al., 2016). However, given the limitations of ToM and other mentalistic approaches in explaining the mechanisms of perspective taking (Barnes-Holmes et al., 2004; Vilardaga, 2009a,b,c; Villatte et al., 2010b), Relational Frame Theory (RFT) researchers proposed an alternative, behavioral theoretical framework to understand perspective taking (McHugh & Stewart, 2012; Vilardaga, 2009a,b,c).

Relational Frame Theory is a modern behavioral account of human language and cognition (Hayes et al., 2001). RFT asserts that perspective taking is dependent on general operant responding, and specifies the relational responses required (Barnes-Holmes et al., 2004). Hence, from an RFT point of view, perspective taking is the ability to respond in accordance with a group of deictic frames, which specify a relation between the perspective of the speaker and the stimulus (Barnes-Holmes et al., 2004; Montoya-Rodríguez et al., 2017). Relational frames of person (I–YOU), place (HERE–THERE), and time (NOW–THEN) are considered fundamental to perspective taking (Barnes-Holmes et al., 2004; Merwin et al., 2020). Understanding of deictic relations emerges through the asking and answering of questions such as, “What are you doing now?”, “What was I doing there?” and “What would you do now if you were me?” These interactions provide opportunities to discuss one’s perspective in relation to the perspective of others (Hayes et al., 2001; Vilardaga, 2009a,b,c; Villatte et al., 2010b). Perspective taking skills develop as people are reinforced for responding in accordance with deictic relations across different environmental contexts (Hayes et al., 2001; Vilardaga & Hayes, 2012).

The RFT model of perspective taking has been studied across different populations, both general and clinical (Montoya-Rodríguez et al., 2017; Vilardaga, 2009a,b,c). One of the earliest studies employed a protocol that targeted the three deictic frames of Here-There, I-You and Now-Then, combined with three levels of relational complexity: simple (“Yesterday I was watching television, today I am reading. What was I doing then?”; McHugh et al., 2004, p. 122), reversed (“I am sitting here on the black chair and you are sitting there on the blue chair. If here was there and there was here. Where would I be sitting?”; McHugh et al., 2004, p. 123), and double reversed (“I am sitting here on the blue chair and you are sitting there on the black chair. If I were you and you were me and if here was there and there was here. Where would I be sitting?”; McHugh et al., 2004, p. 125). Results indicated perspective taking abilities follow a developmental profile, such that performance on the task improved as age increased (McHugh et al., 2004). Significant differences were also found between complexities, where accuracy decreased as complexity increased. These developmental trends are consistent with traditional ToM developmental research (McHugh et al., 2004).

Following this work, new work was proposed to apply RFT to clinical populations (Vilardaga & Plumb, 2006), and other areas of social and clinical concern (Vilardaga et al., 2007; Vilardaga & Hayes, 2010; Vilardaga & Plumb, 2006). New RFT protocols have been used in clinical populations to assess perspective taking skills (Janssen et al., 2014a; Rehfeldt et al., 2007; Villatte et al., 2008; Villatte et al., 2010a; Villatte et al., 2010b). Children with high functioning autism spectrum disorder (ASD; Rehfeldt et al., 2007), adults with social anxiety disorder (Janssen et al., 2014a), and adults with schizophrenia (Villatte et al., 2010b) performed poorer on perspective taking measures compared to their respective controls, especially on reversed items. Taken together, it appears that reversed items are where perspective taking differences become apparent when assessing clinical and non-clinical populations. The ability to accurately interpret reverse deictic relations is important as it is required to infer the intentions of others and is a good predictor of ToM performance in clinical and non-clinical groups (Villatte et al., 2010b).

Both ToM and RFT are models of perspective taking. Hendriks et al. (2016) found strong effects that perspective taking as measured on the ToM tests was positively correlated with the protocol developed by Barnes-Holmes to test perspective taking from an RFT point of view, particularly on the reversed relational perspective takings, i.e., complex perspective taking rather than simple perspective taking. Research has shown that it is possible to improve perspective taking skills in both general and clinical populations, where it has been demonstrated that training respondents on the use of deictic framing led to improvements at testing, which were generalized to ToM performances (Boland et al., 2021; Gilroy et al., 2015; McHugh et al., 2004). Previous reviews of peer-reviewed and conference abstracts regarding the deictic relations approach to typically and atypically developing children and adults have noted the need for further empirical work on applying a deictic relations approach to clinical populations (Montoya-Rodríguez et al., 2017; Vilardaga, 2009a,b,c).

## 1. Borderline Personality Disorder

Studies of empathic perspective taking have found that individuals with Borderline Personality Disorder (BPD) have deficits in this area (Guttman & Laporte, 2000; New et al., 2012; Petersen et al., 2016). The ability to discriminate one’s own perspective from the perspective of another person has implications for understanding others as well as forming healthy relationships (Merwin et al., 2020; Vilardaga & Hayes, 2012). BPD is a multidimensional mental illness characterised by an extensive pattern of instability in affect regulation; impulse control; interpersonal relationships; and self-image (APA, 2013; Lieb et al., 2004). These clinical features lead to marked social, emotional, and functional challenges in this population (APA, 2013; Leichsenring et al., 2011; Skodol et al., 2005). Impaired interpersonal efficacy is a central area of difficulty in this population, with serious health outcomes such as self-injury and suicide, regularly occurring within interpersonal contexts, for example after conflicts or breakups (Lazarus, et al., 2014).

Various theoretical perspectives have aimed to explain the underlying processes in the development and maintenance of interpersonal dysfunction in persons with BPD (Anupama et al., 2018; Petersen et al., 2016). Concepts such as empathy, mentalisation, and affective and cognitive ToM have received most attention within this field. Mentalisation is the process by which we make sense of our own mental state and others’ mental states and may occur within our awareness or outside of our awareness. Mentalisation Based Therapy (MBT) has been shown to be efficacious in treatment of BPD (Fonagy & Bateman, 2008; Malda-Castillo et al., 2019), suggesting an impairment in the ability to mentalise is a feature of the condition (Bales et al., 2015; Bateman & Fonagy, 2010; Paris, 2010). Despite distinctions between mentalisation and ToM (Choi-Kain & Gunderson, 2008; Górska & Marszał, 2014), the terms are often used interchangeably (Colle et al., 2019; Sharp et al., 2011), thereby obscuring any potential differences in these areas for people with BPD. Findings assessing ToM impairments in individuals with BPD are also inconsistent (Anupama et al., 2018; Ghiassi et al., 2010).

In a meta-analysis of ToM disturbances in BPD, Nemeth et al. (2018) found that persons with BPD were more impaired in their overall capacities compared to those in the control condition without BPD. Looking more closely, the ability to decode the mental state of others for those with BPD didn’t differ from the control groups, however, persons with BPD were found to have significantly worse mental state reasoning compared to the control group. Limited research has examined a direct deficit in perspective taking in persons with BPD, and there is no published research using an RFT approach to directly assess perspective taking in persons with BPD.

Despite this limited research examining a direct deficit, there has been a significant amount of research into using a self-report measure, the Interpersonal Reactivity Index (IRI; Davis, 1994), a measure of affective and cognitive dimensions of empathy. With this measure, perspective taking deficits in persons with BPD have consistently been reported (Flasbeck et al., 2017; Guttman & Laporte, 2000; New et al., 2012; Petersen et al., 2016). Petersen et al. (2016) compared IRI self-reported scores of a control group (*n* = 20) to individuals with a diagnosis of BPD (*n* = 19). Results indicated persons with BPD reported significantly lower perspective taking compared to the control group. Similar results were found in a study of women, whereby those with BPD reported lower perspective taking ability on the IRI compared to women with anorexia and the control group (Guttman & Laporte, 2000). Paradoxically, those with BPD are also known for having high levels of empathy and being highly sensitive to the mental states of others (Dinsdale & Crespi, 2013). It has thus been suggested that deficits in mentalisation may not be permanent but rather a transient state activated by interpersonal and/or emotional distress (Fonagy et al., 2011). Taken together, people with BPD self-report impairments in their perspective-taking abilities, but the data is mixed, and further investigation is required to understand the nature of the deficits reported.

## 2. The current study

The current study aimed to examine perspective taking utilising RFT in individuals with BPD through a comparison of clinical and control groups. Specifically, we aimed to assess deictic relational responding in a population of people diagnosed with BPD and a control group using the Deictic Relational Task (DRT), a modified and computerised version of the McHugh et al. (2004) deictic protocol, developed and refined in a series of prior studies that added to a total sample of 372 young adults (Vilardaga et al., 2008, Vilardaga, 2009a,b,c, 2012). In a study examining the association between deictic framing and a psychological trait that has been shown to predict psychosis, social anhedonia, the DRT was found to correlate with social anhedonia after adjusting for age, gender, and empathic concern (Vilardaga et al., 2012). Therefore, given previous evidence of the association of deficits in perspective taking in clinically related phenomena, it was hypothesized that the performance (measured by accuracy and latency) of individuals with a diagnosis of BPD would be poorer on the DRT task compared to the control group. Secondly, using a self-report measure of perspective taking, persons with BPD would score lower compared to the control group. Thirdly, that participants’ performance on the DRT would be correlated with self-reported perspective taking abilities. Finally, we were interested in the hypothesis that greater levels of self-reported overall distress and interpersonal distress in participants with BPD would be associated with poorer performance on the DRT as well as self-reported perspective taking ability.

## 3. Method

### 3.1. Participants

Participants in this study consisted of two groups: an initial clinical group (*n* = 68), with a diagnosis of BPD and a potential comparison control group (*n* = 233).

### 3.2. Procedure

#### 3.2.1. Recruitment

##### 3.2.1.1. Clinical group.

Clinical participants were consumers of a local specialist service for BPD within the public mental health system and were a sub-sample from a larger research study (see Walton et al., 2020; for study protocol and inclusion criteria). Upon referral to the service, clients were allocated to an initial assessment to determine service eligibility. If they met criteria for the service, they were assigned an appointment for a diagnostic interview with a consultant psychiatrist. Following this diagnostic assessment, all clients meeting inclusion criteria were invited to participate in the larger study (Walton et al., 2020). Clients were provided with a full explanation of the procedures and, if willing to participate, signed informed consent was obtained. Participants were informed that participation in the research was voluntary, and they could withdraw from the study at any time without negative consequences.

##### 3.2.1.2. Control group.

All students enrolled in their first year of a Bachelor of Nursing at the University of Newcastle were invited to participate. Invitations and information sheets were sent via e-mail and a notice was placed on the learning management system used at this university. Participation was voluntary, however, students who participated in the study were awarded bonus marks towards their grade for the first-year subject if they had achieved a passing grade for the subject. Following a scheduled lecture for all first-year students, those wanting to participate, were invited to remain in the classroom after the class had finished. A signed consent form was completed prior to participation in the study.

In addition to the measures and tasks specific to this project, The McLean Screening Instrument for Borderline Personality Disorder (MSI-BPD; Zanarini, 2009), was administered to all consenting Bachelor of Nursing participants (*n* = 233) to identify individuals with self-reported BPD traits. Individuals with a score of five or greater on the MSI-BPD (*n* = 62) were screened out. Participants in the control group who reported English to be their second language (*n* = 21) were also screened out.

##### 3.2.1.3. Matching procedure.

Participants in either group who provided incomplete dependent variable measures (*n* = 13) were screened out. For the remaining control group participants (*n* = 137), the researchers sought to identify a matched sample (*n* = 56) as close as possible to the clinical sample (*n* = 56). The matching process was based on the following demographic variables: age, gender identification, ethnicity, marital status, education level, and employment status. This was done manually using Excel spreadsheets and the ‘sort’ function to identify the closest match of the control group to those in the clinical sample. Six of the male participants from the clinical group were unable to be matched to an appropriate peer in the control group and were thus excluded from the analysis.

### 3.3. Data collection

#### 3.3.1. Clinical group

Information regarding data collection is reported in Walton et al. (2020). The study protocol was approved by the Hunter New England Human Research Ethics Committee (Reference Numbers HNEHREC 06/12/13/5.11, 2019/ETH12356) and the University of Newcastle Ethics Committee (Reference Number H-2010-1146).

#### 3.3.2. Control group

Data collection was undertaken within the first week of the first semester. This was to ensure ease of access to the control population and minimal disruption to student timetables. The study protocol was approved by the University of Newcastle Ethics Committee (Reference Number H-2014-0037).

### 3.4. Measures and tasks

#### 3.4.1. Demographics

Participant demographic items included: date of birth; age; gender identification (Male; Female); race and ethnicity (Aboriginal and/or Torres Strait Islander; White Caucasian Australian; Other); if English was their first language (Yes; No); marital status (Married; De-facto; Single; Divorced); level of education completed (less than twelve years of high school; High School graduate; TAFE course; Some university; University Graduate); and employment status (Employed; Unemployed).

#### 3.4.2. Deictic relational task

The Deictic Relational Task (DRT; Vilardaga, Jeffcoat, Levin, & Hayes, 2011, July; 2012) is a behavioral measure designed to assess perspective-taking in an adult population. It involves a series of hypothetical scenarios. Items differed based on the deictic relation which they were testing (Here-There, I-You or Now-Then). Only reversed deictic relation items were included, as they have been shown to discriminate between control and clinical group perspective taking ability (Villatte et al., 2012). Across deictic groups, items also differed on affective and cognitive dimensions. Half of the items asked participants how they would feel in a scenario (e.g., “This morning, you heard some good news and now, you are hearing some bad news. If now was this morning, how would you be feeling?”), while the other half targeted cognitive aspects of perspective taking (e.g., “I am facing a wall and you are facing a mirror. If the wall was the mirror, what would I see?”).

The DRT consisted of a battery of 48 scenarios, each of them with a corresponding item and two response options. The items and corresponding response options were presented on a computer using macros in Microsoft PowerPoint, with the program recording responses directly onto the computer. The ordering of the presentation of scenarios was randomised. If errors were made, participants were not provided with corrective feedback. To accommodate for deficits in attention and working memory, each scenario was presented for as long as the participant needed.

Both accuracy rates and (correct) response latency were recorded (with longer response times predicted to reflect poorer performance). More frequent errors on the task combined with a slower response time indicate lower ability to switch perspective taking. The task took approximately 10–12 min to complete. Questions were of neutral content and were not expected to be distressing for participants.

#### 3.4.3. Interpersonal Reactivity Index

The Interpersonal Reactivity Index (IRI; Davis, 1980) is a multidimensional self-report scale of empathy. The Perspective Taking domain (comprised of 7 items) was used in the present study and measures empathy as a cognitive process. It evaluates the tendency to understand the psychological view of others, with items such as “I try to look at everybody’s side of a disagreement before I make a decision”. Responders are asked to indicate their level of agreeance with each item on a five-point Likert scale (‘does not describe me well’ to ‘describes me very well’). High summary scores indicate greater perspective taking. Psychometric properties of the IRI indicate appropriate internal consistency (Davis, 1994), with the Perspective Taking subscale yielding Cronbach Alphas between 0.71 and 0.79 (Davis, 1980; Pulos et al., 2004).

#### 3.4.4. Distress rating scale

Participants in the clinical group were asked to complete two items: “How would you rate your level of distress at this moment (from 0 to 10)?”; and “How much of this distress (from 0 to 10) is to do with a relationship with someone who is important to you?” Zero indicated no current distress or no distress related to a relationship and 10 indicated maximum distress or the distress was very much related to a relationship. The rating scale was specifically designed for the purposes of the study and was based on the Subjective Units of Distress Scale (SUDs; Wolpe, 1990). In this study, the scale was used to assess whether current distress in people with BPD, particularly interpersonal distress, would have a mediating effect on the completion of interpersonal measures such as the DRT and IRI.

### 3.5. Data analysis

With respect to statistical power, a sample size of 64 participants per group was considered desirable, which would provide 80% power to detect population differences of the order of 0.50 of a standard deviation (a moderate effect size), using 0.05 level two-tailed significance tests; by comparison, the obtained sample size (*n* = 56 per group) provided 75% power to detect such differences.

Descriptive statistics, including means, standard deviations, and ranges, were examined for all measures. Chi-squared tests were used to confirm that the clinical and control groups were appropriately matched and not statistically different from each other. Planned comparisons analysis, within the context of 2 × (3 × 2) repeated measures analysis of variance (ANOVA), was conducted for both accuracy and latency dependent measures; that is, a *group* (between subjects factor: control vs. clinical group) by *deictic frame* (within subjects factor: Here-There, I-You, Now-Then) by *item type* (within subjects factor: affective vs. cognitive) design. Between subjects ANOVA was used to assess whether individuals with BPD self-reported deficits in perspective taking abilities on the IRI compared to the control group. The DRT planned comparisons analyses were also re-run within the clinical group, with additional covariates examining whether overall and interpersonal distress in persons with BPD mediated DRT performance. Pearson’s correlation was also used to assess relationships between performance on the DRT and self-reported perspective taking abilities.

## 4. Results

Table 1 displays demographic characteristics by experimental group. Chi-squared tests showed groups were appropriately matched on all demographics except for employment status and education status. For employment status, 76.8% of the control group were employed compared to 43.6% of the clinical group, *X*^2^ = 12.74, p < 0.001. This is consistent with previous research that has shown people with BPD to be less likely to be employed compared with a sample of people without BPD (Sansone & Sansone, 2012). Education status (*X*^2^ = 17.78, p < 0.001) was a mixed picture, where a larger percentage of the clinical sample (21.4%) than the control group (5.4%) had completed less than 12 years of education, but also a greater percentage of the clinical sample (17.9%) were University graduates compared to the control group (7.1%). The control group were all nursing students, hence, some of the differences in education are a function of recruitment source.

### 4.1. Deictic relational responding task

#### 4.1.1. Accuracy

Proportion of responses answered accurately were calculated for each participant. Rates of overall accuracy (across the 48 DRT items) were identical between groups (Control *M* = 0.88, *SD* = 0.10; Clinical *M* = 0.88, *SD* = 0.11, *p* = 0.969). Table 2 shows the descriptive statistics for DRT accuracy by group, deictic frame, and item type. Across groups, participants responded more accurately to the I-You deictic frame (*M* = 0.92, *SE* = 0.013) compared to Here-There (*M* = 0.85, *SE* = 0.013) [F_(1,110)_ = 21.66, *p* < 0.001, $\eta_{\text{p}}^{2}$ (partial eta-squared) = 0.165] and Now-Then (*M* = 0.87, *SE* = 0.015) [F_(1,110)_ = 5.21, *p* = 0.024, $\eta_{\text{p}}^{2}$ = 0.045]. The magnitude of the I-You versus Here-There difference in accuracy tended to be more marked in the Control group (*M* = 0.93 vs 0.83) compared with the Clinical group (*M* = 0.90 vs 0.87) [interaction contrast: F_(1,110)_ = 5.60, *p* = 0.020, $\eta_{\text{p}}^{2}$ = 0.048], but this difference was not significant when employment and education status were included as covariates [interaction contrast, with covariates: F_(1,110)_ = 3.14, *p* = 0.079, $\eta_{\text{p}}^{2}$ = 0.029]. While there was no main effect for item type, there were statistically significant deictic frame by item type interaction contrasts. As seen in Table 2, responses to Here-There deictic frames were less accurate for affective versus cognitive item types (*M* = 0.82 vs 0.88), whereas the opposite was the case for the I-You deictic frame (*M* = 0.93 vs 0.91) [interaction contrast: F_(1,110)_ = 7.13, *p* = 0.009, $\eta_{\text{p}}^{2}$ = 0.061] and the Now-Then deictic frame (*M* = 0.89 vs 0.86) [interaction contrast: F_(1,110)_ = 9.84, *p* = 0.002, $\eta_{\text{p}}^{2}$ = 0.082].

To assess the potential influence of overall distress and interpersonal distress on DRT accuracy performance of the clinical group, the planned comparisons analyses (deictic frame by item type) were re-run with the distress measures included as covariates. The were no significant main effects or interactions involving the distress measures, suggesting that they did not moderate DRT accuracy.

#### 4.1.2. Latency

Aggregate reaction times for correct responses (across the 48 DRT items) were similar between groups (Control *M* = 14.54 s, *SD* = 4.12; Clinical *M* = 14.12 s, *SD* = 3.97, *p* = 0.593). Table 3 shows the descriptive statistics for DRT latency (for correct responses) by group, deictic frame, and item type. In this instance, a very clear pattern was evident in the planned comparisons analyses, with differences between deictic frames, and between item types, but no significant group or interaction effects. Across groups, participants were quickest at correctly responding to I-You (*M* = 12.88, *SE* = 0.38) deictic frames compared to Now-Then (*M* = 14.11, *SE* = 0.43) [F_(1,109)_ = 26.70, *p* < 0.001, $\eta_{\text{p}}^{2}$ = 0.197] and Here-There (*M* = 16.03, *SE* = 0.49) frames [F_(1,109)_ = 115.98, *p* < 0.001, $\eta_{\text{p}}^{2}$ = 0.516]. Now-Then frames were also significantly faster than Here-There frames [F_(1,109)_ = 34.14, *p* < 0.001, $\eta_{\text{p}}^{2}$ = 0.239]. Participants were also quicker at correctly responding to cognitive items (*M* = 13.64, *SE* = 0.41) when compared to affective items (*M* = 15.04, *SE* = 0.42) [F_(1,109)_ = 41.87, *p* < 0.001, $\eta_{\text{p}}^{2}$ = 0.278].

Once again, to assess the influence of overall distress and interpersonal distress on DRT accurate latency performance in the clinical group, the planned comparisons analyses (deictic frame by item type) were re-run with the distress measures included as covariates. Inclusion of these covariates had minimal impact, and the deictic frame and item type differences noted above were evident once again.

### 4.2. Interpersonal Reactivity Index (IRI) – perspective taking subscale

An independent samples *t*-test was conducted to compare self-reported perspective taking abilities between control and clinical groups. There was a significant difference in the self-reported perspective taking scores between groups, with the control group reporting greater perspective taking abilities (*M* = 18.46, *SD* = 4.90) than the clinical group (*M* = 13.93, *SD* = 6.44) [*t*_(110)_ = 4.20, *p* < 0.001]. Pearson’s correlations were used in the clinical group to assess the relationship between self-reported perspective taking ability on the IRI and level of self-reported distress. No correlation was found between IRI and overall distress (*r* = 0.183, *n* = 56, *p* = 0.177) or between IRI and interpersonal distress (*r* = −0.074, *n* = 56, *p* = 0.587).

### 4.3. Association between perspective taking measures

Separate Pearson’s correlations were used to assess whether a positive relationship existed across groups between the self-reported perspective taking score on the IRI and performance (accuracy and latency) on the DRT, as summarised in Table 4. Statistically significant, but modest, positive correlations were found between the perspective taking score and latency for I-You affective items and for Here-There and Now-Then cognitive items.

## 5. Discussion

The present study is the first to use a RFT approach to directly examine deficits in perspective taking in persons with BPD. Our aim was to identify the core functional processes underlying deficits reported in the Theory of Mind and Mentalisation literature, in line with previous RFT research on schizophrenia, social anhedonia, and autism. Contrary to our hypothesis, perspective taking deficits measured on the DRT were not observed in persons with BPD when compared to a control group. Similarly, overall distress and interpersonal distress were not found to have a significant impact on DRT performance for persons with BPD. However, in line with our prediction, persons with BPD self-reported lower perspective taking abilities compared to a control group. Finally, self-reported perspective taking ability was also positively correlated with latency for accurate responses within some of the item groupings (see Table 4), with slower reaction times on accurate responses associated with greater self-reported perspective taking ability.

### 5.1. Perspective taking between groups

The finding that persons with BPD did not display a significant difference from the control group may be explained in several ways. Firstly, the results may be to do with the task itself. With high rates of overall accuracy and relatively fast reaction times across the groups, reversed deictic frames may have been of low difficulty and thus no deficit could be detected. Future research should consider using double reversed deictic frames in assessing perspective taking ability in BPD. The inconsistency between DRT performance and self-reported perspective taking ability may partially be explained by the fact that the IRI, as a self-reported measure, captures psychological processes that go above and beyond deictic relations themselves, such as family or individual self-rules, cultural norms, and/or social desirability.

Considering this idea further, the DRT may measure more fundamental aspects of perspective-taking and thus not capture deficits in more complex abilities that build on these foundational skills, such as mentalisation and ToM. Colle et al. (2019) discuss inconsistent findings in the literature regarding whether people with BPD have deficits in their ToM Capacity. They suggest that these inconsistencies in terms of ToM impairments in individuals with BPD may be a result of the varying methodologies used in the research. Previous research has found that individuals with BPD were more likely to make ToM-based errors on ecologically valid tasks that had greater complexity than simpler tasks (Preiβler, et al., 2010) leading Roepke et al. (2013) to argue that more complex tasks may be necessary to tease out ToM deficits in individuals with BPD.

Aside from the task, the construct of perspective taking itself is complex. Kavanagh et al. (2020) suggest that reviewing the development and clinical literature on perspective taking and ToM highlights the challenge of distinguishing perspective taking from other broader cognitive constructs. Sabbagh (2004) identified 2 processes of ToM: 1) the ability to detect and discriminate cues in the immediate social environment, i.e., to decode the mental state of others and 2) to make inferences about those cues, i.e., reason about the mental state of others. Nemeth et al. (2018) found that the ability of persons with BPD to decode the mental state of others didn’t differ from a control group of those without BPD, but their mental state reasoning was worse. The DRT task may assess decoding but not mental state reasoning which may explain why we didn’t find any differences. Similarly, Davis et al. (2004) summarise 4 steps involved in perspective taking highlighting the complexity of the construct.

The first deictic protocol was originally designed to assess the developmental profile of relational responding and to establish deictic relations when found to be absent or deficient (McHugh et al., 2004), and questions have been raised regarding the suitability of generalising a protocol designed for children to an adult population (Hussey et al., 2014; Kavanagh et al., 2019). Thus, the DRT may measure a basic skill of perspective-taking that is intact for those with BPD, and deficits may appear with more complex skills. The lack of specificity in the literature may perpetuate the belief that persons with BPD have difficulties in perspective taking, rather than a deficit in mentalisation or ToM. Using a contextual behavioral model with RFT at its base, Vilardaga and Hayes (2012) proposed a conceptual model of pathological altruism that hypothesized that it is the combination of a deictic framing repertoire (perspective taking), the transformation of functions of deictic relations (empathy), and contextual control over those deictic relational functions (psychological flexibility) that is at the core of a more stable and effective sense of self. This process, also coined as the ‘flexible connectedness model’ was tested in social anhedonia (Vilardaga et al., 2012) and generalized prejudice (Levin et al., 2016; Vilardaga et al., 2014). This conceptual model aligns with prior literature in ToM and mentalisation with regards to the fact that perspective taking is a complex cognitive and affective process that interacts with other related psychological processes, above and beyond perspective ability itself.

In support of this theory are the results showing that higher latencies (i.e. poorer performance) were associated with higher IRI scores for some of the item categories. This suggests that the construct measured by the IRI and by the DRT were overlapping but also distinct. That is, the IRI may mostly be capturing the ability of these participants to transform the functions of these deictic relations into actual emotional understanding between people, and it may also be the case that considering responses for longer is an important component to transform the functions of these deictic relations (i.e., accurate empathy in relationships).

It is also possible that individuals with BPD may under report their perspective taking abilities. This may be due to an added and unmeasured process of experiential avoidance (i.e., psychological inflexibility), which may have a parallel in what has been described in the literature as a self-critical cognitive style (Feliu-Soler et al., 2017; Gilbert & Irons, 2005). Traits such as self-invalidation and self-criticism are over-represented in individuals with BPD compared to the general population (Donald, et al., 2019; Feliu-Soler et al., 2017; Zeigler–Hill & Abraham, 2006). With a reduced sense of self-confidence and self-esteem, a very basic deictic relational task like the DRT, and a self-report measure, like the IRI, may not accurately capture these additional processes among individuals with BPD.

Furthermore, individuals may under-report their abilities because of the impact of interpersonal dysfunction and emotional regulation difficulties, which are core features of BPD and have a strong correlation with experiential avoidance. That is, those with BPD may “work backwards” and think that their perspective taking abilities must be poor to lead to the dysfunction they experience and may indeed receive frequent and salient feedback about unsuccessful attempts to take the perspective of another. Further, 1) individuals with BPD may have the ability to perspective take, yet may avoid doing so to prevent suffering that may result from attuning to the distress of others (Hussey et al., 2014); 2) those with BPD are more likely to experience abusive relationships where blame for dysfunction is misattributed to them (Laporte & Guttman, 2001), leading them to believe that they have a deficit in this area; and 3) those with BPD may be hypersensitive and dysfunction may result from interpreting cues at a higher intensity than intended (Sharp & Vanwoerden, 2015). These alternative explanations would mean that their basic perspective taking abilities are intact but that deficits still occur when relating to others. Taken together, those with BPD may have scored lower on the IRI without any actual deficits in their fundamental perspective taking abilities, which is consistent with the known paradox where those with BPD are regarded as being highly empathic (Dinsdale & Crespi, 2013), and in line with the flexible connectedness model described above and in the contextual behavioral science literature.

### 5.2. Distress as a mediator of perspective taking performance

Overall distress and interpersonal distress within the clinical group were not significantly related to deictic relational responding performance (accuracy or latency), nor were they significantly related to overall self-reported perspective taking ability. This suggests that interpersonal and overall distress may require additional contextual cues that evoke distressing self or other deictic functions, such as emotional concern about others, self-blame, or other negative functions. In addition, the testing environment was relatively calm and unlikely to have triggered interpersonal distress, and so deficits may not have been activated. Fonagy and Luyten (2009) suggest that mentalising capacity in persons with BPD may only be compromised in situations where their attachment system is activated. Hence, ToM capacity may be maintained in situations that are emotionally neutral and only break down in situations of emotional arousal associated with attachment threat (Vaskinn et al., 2015). Thus, the protocol employed in this study may not be suitable for capturing the deficits reported in those with BPD, which reflects previous criticism of the use of DRT tasks in clinical populations (Kavanagh et al., 2019). Future research that induces distress via the use of more direct contextual cues that evoke the transformation of specific deictic relational functions may find an impact on perspective taking performance.

### 5.3. Overall DRT findings

Across groups, individuals were faster at correctly responding to I-You deictic frames compared to Here-There and Now-Then frames. This contrasts with previous DRT literature where no statistical difference in latencies by deictic frame were observed (Villatte et al., 2008, 2010b), although it should be noted that the DRT was a new version of the task than had been used previously. As found in previous research, participants were more accurate on I-You deictic frames than Here-There or Now-Then deictic frames (McHugh et al., 2004; Villatte et al., 2010b). Increased accuracy and quicker reaction times to I-You deictic frames may suggest that participants find responding to allocentric perspective taking I-You deictic frames easier compared to egocentric Here-There and Now-Then frames (Villatte et al., 2008). Similarly, across groups, individuals were quickest at correctly responding to cognitive deictic frame items compared to affective. This may suggest that understanding the emotional perspective of others is a more difficult task or requires a different process compared to understanding the cognitive mental state of others (Bradford et al., 2015). This finding can be also interpreted by arguing that deictic items linked to affective contextual cues are more likely to trigger an experiential avoidance response that leads to longer latencies in the DRT task, which is consistent with the flexible connectedness model. To our knowledge, it is the first time that a distinction between cognitive and affective deictic framing is demonstrated in the RFT literature.

### 5.4. Strengths, limitations and future directions

This study is the first of its kind to use an RFT approach to assess perspective taking in persons with BPD. Current literature regarding perspective taking in this clinical population lacks clarity in its definition of related, yet distinctly different, theoretical concepts such as mentalisation and ToM. Relatedly, ToM literature has yielded inconsistent results regarding persons with BPD, which may be a result of the varying methodologies utilised (Colle et al., 2019). Utilising an RFT approach allowed for an assessment of perspective taking in persons with BPD from a functional contextual point of view. Although we did not find differences between participants with BPD and control participants on deictic framing, our study shed new light on the distinction between cognitive and affective deictic framing and suggested the need to explore new conceptual frameworks in contextual behavioral science (e.g., the flexible connectedness model) to further improve our understanding of these complex processes in clinical populations. We believe that these complex models should be further explored in future RFT research on schizophrenia, autism, and different developmental stages in non-clinical populations. In terms of our understanding of BPD and perspective taking, future research should explore use of more complex deictic relational tasks that more deliberately incorporate contextual cues to evoke negative affective responses, as well as flexible patterns of responding that would contribute to gain psychological distance from them (i.e., specific ACT metaphors) among those with BPD. Future research is needed to clarify concepts such as ToM, mentalisation and perspective taking and their application to persons with BPD.

## 6. Conclusions

The present study found that there was no difference in an objective measure of perspective taking in persons with BPD compared to the general population, and that overall distress and interpersonal distress did not have a role in perspective taking abilities in persons with BPD. In line with previous research, however, a significant difference between groups was found in a self-report measure of perspective taking in persons with BPD, suggesting a discrepancy between perceived and measured ability. The lack of significant difference between groups may be partially explained by low difficulty of DRT items, deficits in mentalisation rather than perspective taking, and not adequately capturing the role of other RFT processes that are conceptually linked to deictic framing ability. Consequently, further research is required to understand the underlying processes of the development and maintenance of interpersonal dysfunction in the population, enabling treatment to be directly tailored to address these deficits.

## Figures and Tables

**Table 1:** Demographics of study participants (*N* = 112).

Variable	Control *n* = 56		Clinical *n* = 56	
	%	*n*	%	*n*
Age, years (range 18–55 *M* = 25.56, *SD* = 7.30)^a^				
18–24	53.6	30	50	28
25–31	28.6	16	35.7	20
32–39	12.5	7	8.9	5
40+	5.4	3	5.4	3
Gender identification				
Male	17.9	10	17.9	10
Female	82.1	46	82.1	46
Ethnicity				
White Caucasian Australian	94.6	53	87.5	49
Aboriginal and/or Torres Strait Islander	3.6	2	3.6	2
Other	1.8	1	8.9	5
Marital Status^b^				
Married	13.0	7	8.9	5
De-Facto	22.2	12	11.1	9
Single	59.3	32	69.6	39
Divorced	5.6	3	5.4	3
Highest education level achieved				
Less than Year 12	5.4	3	21.4	12
High School Graduate	32.1	18	12.5	7
TAFE Course	23.2	13	33.9	19
Some University	32.1	18	14.3	8
University Graduate	7.1	4	17.9	10
Employment Status^c^				
Employed	76.8	43	43.6	24
Unemployed	23.2	13	56.4	31

a Control Age range 18–51 *M* = 25.54 *SD* = 7.12; Clinical Age: range 18–55 *M* = 25.59 *SD* = 7.45.

b Control *N* = 54 due to missing information.

c Clinical *N* = 55 due to missing information.

**Table 2:** DRT accuracy (proportion correct) - descriptive statistics (*n* = 112).

	Control		Clinical	
	M	SD	M	SD
Affective				
Here-There	0.79	0.20	0.85	0.19
I-Yo	0.95	0.15	0.90	0.15
Now-Then	0.89	0.18	0.88	0.19
Cognitive				
Here-There	0.86	0.16	0.89	0.14
I-You	0.91	0.18	0.90	0.15
Now-Then	0.86	0.16	0.85	0.20

**Table 3:** DRT latency for correct responses (in seconds) - descriptive statistics (*n* = 112).

	Control^a^		Clinical	
	*M*	*SD*	*M*	*SD*
Affective				
Here-There	16.81	5.68	16.26	4.83
I-You	13.95	4.65	13.79	4.92
Now-Then	15.20	5.69	14.22	4.28
Cognitive				
Here-There	15.53	6.41	15.52	6.07
I-You	12.13	3.66	11.65	4.06
Now-Then	13.76	5.06	13.26	4.74

a One of the Control participants had none of the I-You items correct.

**Table 4:** Pearson’s correlation between DRT performance and IRI perspective taking score (*N* = 112).

	Accuracy		Latency	
	r	P	r	p
Affective				
Here-There	−0.005	0.956	0.131	0.169
I-You	0.108	0.257	0.259^b^	0.006
Now-Then	0.102	0.286	0.181	0.056
Cognitive				
Here-There	0.042	0.662	0.218^a^	0.021
I-You	0.039	0.686	0.150	0.115
Now-Then	0.160	0.092	0.206^a^	0.029

a *p* < .05 (2-tailed).

b *p* < .01 (2-tailed).
